# Urinary C‐peptide creatinine ratio detects absolute insulin deficiency in Type 2 diabetes

**DOI:** 10.1111/dme.12222

**Published:** 2013-06-12

**Authors:** S. V. Hope, A. G. Jones, E. Goodchild, M. Shepherd, R. E. J. Besser, B. Shields, T. McDonald, B. A. Knight, A. Hattersley

**Affiliations:** ^1^ Department of GeriatricsRoyal Devon and Exeter NHS Foundation TrustExeterUK; ^2^ NIHR Exeter Clinical Research FacilityExeterUK; ^3^ Department of BiochemistryRoyal Devon and Exeter NHS Foundation TrustExeterUK

## Abstract

**Aims:**

To determine the prevalence and clinical characteristics of absolute insulin deficiency in long‐standing Type 2 diabetes, using a strategy based on home urinary C‐peptide creatinine ratio measurement.

**Methods:**

We assessed the urinary C‐peptide creatinine ratios, from urine samples taken at home 2 h after the largest meal of the day, in 191 insulin‐treated subjects with Type 2 diabetes (diagnosis age ≥45 years, no insulin in the first year). If the initial urinary C‐peptide creatinine ratio was ≤0.2 nmol/mmol (representing absolute insulin deficiency), the assessment was repeated. A standardized mixed‐meal tolerance test with 90‐min stimulated serum C‐peptide measurement was performed in nine subjects with a urinary C‐peptide creatinine ratio ≤ 0.2 nmol/mmol (and in nine controls with a urinary C‐peptide creatinine ratio >0.2 nmol/mmol) to confirm absolute insulin deficiency.

**Results:**

A total of 2.7% of participants had absolute insulin deficiency confirmed by a mixed‐meal tolerance test. They were identified initially using urinary C‐peptide creatinine ratio: 11/191 subjects (5.8%) had two consistent urinary C‐peptide creatinine ratios ≤ 0.2 nmol/mmol; 9 of these 11 subjects completed a mixed‐meal tolerance test and had a median stimulated serum C‐peptide of 0.18 nmol/l. Five of these 9 had stimulated serum C‐peptide <0.2 nmol/l and 9/9 subjects with urinary C‐peptide creatinine ratio >0.2 had endogenous insulin secretion confirmed by the mixed‐meal tolerance test. Compared with subjects with a urinary C‐peptide creatinine ratio >0.2 nmol/mmol, those with confirmed absolute insulin deficiency had a shorter time to insulin treatment (median 2.5 vs. 6 years, *P*=0.005) and lower BMI (25.1 vs. 29.1 kg/m^2^, *P*=0.04). Two out of the five patients with absolute insulin deficiency were glutamic acid decarboxylase autoantibody‐positive.

**Conclusions:**

Absolute insulin deficiency may occur in long‐standing Type 2 diabetes, and cannot be reliably predicted by clinical features or autoantibodies. Absolute insulin deficiency in Type 2 diabetes may increase the risk of hypoglycaemia and ketoacidosis, as in Type 1 diabetes. Its recognition should help guide treatment, education and management. The urinary C‐peptide creatinine ratio is a practical non‐invasive method to aid detection of absolute insulin deficiency, with a urinary C‐peptide creatinine ratio > 0.2 nmol/mmol being a reliable indicator of retained endogenous insulin secretion.

## Introduction

Most older patients with diabetes have Type 2 diabetes, which is typically a disease where endogenous insulin persists. Progressive β‐cell dysfunction occurs in Type 2 diabetes 1, but it is unclear if this leads to absolute insulin deficiency. By contrast, in Type 1 diabetes absolute insulin deficiency is usual outside the initial ‘honeymoon period’, the period soon after diagnosis when some residual β‐cell function may persist 5.

Some patients may present clinically later in life as having Type 2 diabetes, but have the autoimmune destructive process as seen in Type 1 diabetes. These patients can be recognised by pancreatic autoantibodies, known as latent autoimmune diabetes of adulthood (LADA) 6. People with LADA may develop absolute insulin deficiency 7. In practice, however, autoantibody levels are rarely measured in patients presenting with adult‐onset diabetes: a clinical diagnosis of Type 2 diabetes is usually made and seldom revisited, and so later subsequent development of absolute insulin deficiency is rarely suspected or tested for.

Absolute insulin deficiency in patients with Type 2 diabetes is likely to carry similar risks to those associated with Type 1 diabetes, such as fluctuant blood glucose levels, high hypoglycaemia risk and diabetic ketoacidosis 11. The patient with Type 2 diabetes, however, is unlikely to be offered a similar level of education to deal adequately with these, such as the Dose Adjustment for Normal Eating (DAFNE) programme 12. Frail older people, in particular, may be ill‐equipped to cope with such complications, with less functional reserve both physically and cognitively, and in terms of their social support. The development of absolute insulin deficiency in Type 2 diabetes will alter treatment: oral hypoglycaemic agents (especially sulphonylureas) will not be effective, the newer agents, e.g. glucagon‐like peptide (GLP)‐1 receptor analogues and dipeptidyl peptidase (DPP)4 inhibitors, are not suitable, and the most appropriate insulin regimen may be basal‐bolus rather than background long‐acting insulin. With an estimated 870 000 people with insulin‐treated Type 2 diabetes in the UK, the development of absolute insulin deficiency in even a small proportion could have significant impact on both individuals and society.

Endogenous insulin levels are rarely measured in routine clinical practice, even in secondary care, owing to practical limitations, including the need for rapid laboratory analysis of blood tests. The majority of patients with Type 2 diabetes are cared for in primary care where this is even less practical. Recently, a simple urine test, the urinary C‐peptide creatinine ratio (UCPCR) 13, has been shown both in Type 1 diabetes and Type 2 diabetes, to be excellently correlated with the ‘gold standard’ measure of endogenous insulin secretion, the formal mixed‐meal tolerance test (MMTT), and a sensitive and specific test for absolute insulin deficiency 5. The UCPCR test has the advantages of being widely available, and stable at room temperature for 3 days, so offering the potential for widespread non‐invasive testing which may be particularly useful for a more frail, older population. The aim of the present study was to use the UCPCR to test for absolute insulin deficiency in older people with insulin‐treated Type 2 diabetes.

## Methods

### Subjects

A total of 191 insulin‐treated subjects with Type 2 diabetes (clinical diagnosis of Type 2 diabetes, diagnosis at ≥ 45 years of age, insulin treatment not started within 1 year of diagnosis) were recruited from primary care at the time of their routine retinal screening appointment, and written consent was obtained for participation in the study. Baseline data collected included duration of diabetes, current treatment, BMI and most recent HbA_1c_ concentration.

### Urine collection and analysis

Participants were asked to provide an initial urine sample, collected at home, 2 h after their largest meal of the day. The urine sample was collected in a standard mid‐stream urine boric acid‐containing specimen pot, and returned by post to the routine pathology laboratories for UCPCR analysis. UCPCR ≤ 0.2 nmol/mmol is equivalent to a stimulated serum C‐peptide (sSCP) of 0.2 nmol/l in an MMTT 15, representing an absence of clinically significant insulin secretion 11. This level is associated with unstable glycaemia, increased risk of hypoglycaemia and microvascular complications (as well as absolute insulin requirement) in Type 1 diabetes 11.

All patients identified as insulin‐deficient were asked to provide a repeat sample to confirm their initial result, as were a random group of those with a UCPCR >0.2 nmol/mmol.

### Mixed‐meal tolerance test

In those patients with consistent UCPCR results ≤0.2 nmol/mmol, we performed a formal MMTT with their insulin excluded, to confirm the absolute insulin deficiency 5. A comparison group of age‐matched participants with UCPCR >0.2 nmol/mmol also underwent the standardized MMTT 17. In brief, subjects fasted from midnight, and omitted their morning medications including insulin. Fasting serum and urine samples were taken before participants consumed 6 ml/kg Ensure Plus HP (Abbott Laboratories, Abbott Park, IL, USA). A blood sample for sSCP was taken 90 min later, and a urine sample for UCPCR at 2 h. As above, a sSCP concentration of <0.2 mmol/l was used to represent absolute insulin deficiency 5.

### Sample analysis

Urine and serum samples were analysed for C‐peptide using an electrochemiluminescence immunoassay (intra‐assay coefficient of variation <3.3%; interassay coefficient of variation <4.5%) on a Roche Diagnostics E170 analyser (Roche, Mannheim, Germany) by the biochemistry department at the Royal Devon and Exeter NHS Foundation Trust. Urine creatinine was analysed on the Roche P800 platform using creatinine Jaffé reagent (standardized against isotope dilution mass spectrometry) to obtain a urinary C‐peptide creatinine ratio. Blood samples for all patients completing the MMTT were analysed for glutamic acid decarboxylase (GAD)65 and islet antigen 2 (IA2) autoantibodies, using the Biokit automated Elisa System (BEST 2000; Biokit, Barcelona, Spain) following the manufacturer's instructions. The cut‐offs used were those based on the 99th centile for 500 individuals without diabetes; for GAD65 the reference‐positive value was >64 units/ml, for IA2 the reference‐positive value was >15 units/ml.

### Data analysis

**Figure 1:**
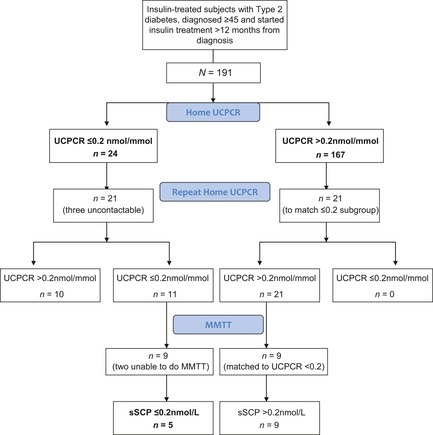
Flow of participants through the study. UCPCR, urinary C‐peptide creatinine ratio; MMTT, mixed‐meal tolerance test; sSCP, stimulated serum C‐peptide.

The data were not normally distributed, and so are presented as medians and interquartile ranges (IQRs). Results were analysed primarily by comparing the clinical characteristics of those with confirmed absolute insulin deficiency on MMTT and those with endogenous insulin secretion, using Mann–Whitney *U*‐ and chi‐squared tests (using Predictive Analytic Software: PASW 17.0). The full group of 167 participants with an initial home UCPCR >0.2 nmol/mmol was used to represent those with significant insulin secretion, given the consistency of repeat UCPCR and MMTT results in subgroups drawn from these (see Results and Fig. 1).

Ethics approval was obtained from the Southwest Research Ethics Committee.

## Results

A total of 191 participants, with a median (IQR) age 73.5 (67–78) years and of whom 37% were women, provided an initial urine sample for UCPCR measurement. They had a median (IQR) age at diagnosis of 58 (50–65) years, duration of diabetes of 13.5 (9–19) years, and BMI at recruitment of 29 (25.9–33.54) kg/m^2^. Their median (IQR) time to insulin treatment from diagnosis was 6 (3.5–11) years.

Figure 1 shows the flow of patients through the study. Of the 191 participants screened, 24 (12.5%) had UCPCR ≤ 0.2 nmol/mmol. Of these, 21 provided a repeat sample, and 11/188 (6% of the whole cohort) had two consistent UCPCR results of ≤ 0.2 nmol/mmol.

Table 1 shows the MMTT results of the two groups selected on the basis of their UCPCR. These two groups were similar in age, duration of diabetes, time to insulin from diagnosis, and BMI. As expected the sSCP concentration was lower in those with a low UCPCR than in those with a high UCPCR (median 0.18 vs. 2.0 nmol/l, respectively, *P* = 0.002). Five of the nine participants with a low UCPCR had a sSCP of <0.2 nmol/l, representing absolute insulin deficiency 18, in contrast to none with a high UCPCR had a sSCP <0.2 nmol/l. This suggests a minimum prevalence of absolute insulin deficiency in insulin‐treated Type 2 diabetes of 3% [5/186, excluding the five subjects who were unable to provide repeat urine samples or participate in the MMTT (Fig. 1)].

**Table 1:** Urinary C‐peptide creatinine ratios and stimulated serum C‐peptide values in nine subjects with two home UCPCRs of ≤0.2 nmol/mmol, compared with nine matched subjects with two home UCPCRs of >0.2 nmol/mmol

	UCPCR ≤0.2 nmol/mmol	UCPCR >0.2 nmol/mmol	*P*
UCPCR (home), nmol/mmol	<0.02 (<0.02–0.2)	1.7 (0.8–7.1)	<0.001
UCPCR (MMTT) nmol/mmol	0.07 (<0.02–0.7)	2.6 (1.9–5.6)	0.001
fSCP, nmol/l	0.13 (0.08–0.35)	0.59 (0.45–0.88)	0.003
sSCP, nmol/l	0.18 (0.08–0.64)	2.0 (1.53–2.52)	0.002

Data are shown as median values (interquartile range).

UCPCR, urinary C‐peptide creatinine ratio; MMTT, mixed‐meal tolerance test; fSCP, fasting serum C‐peptide; sSCP, stimulated serum C‐peptide.

Notably, the UCPCR results obtained in both groups were substantially higher after the MMTT than after the home meal. For those four patients with two home UCPCRs ≤ 0.2 nmol/mmol but an sSCP >0.2 nmol/l, the post‐MMTT UCPCR results were also >0.2 nmol/mmol. This suggests the MMTT provided more β‐cell stimulation than did the meals consumed at home.

The five patients with confirmed absolute deficiency on MMTT had a lower BMI (BMI 25.1 vs. 29.1 kg/m^2^, *P*=0.04), and commenced insulin treatment more rapidly after diagnosis (2.5 vs. 6 years, *P*=0.005), although there was substantial overlap for both these measures between those with (*n*=5) and without (*n*=167) absolute insulin deficiency. There was no difference in age of diagnosis, duration of diabetes, glycaemic control or insulin dose (Table 2).

**Table 2:** Clinical characteristics of those with absolute insulin deficiency as confirmed by a mixed‐meal tolerance test, vs those with endogenous insulin secretion (urinary C‐peptide creatinine ratio >0.2 nmol/mmol)

	Absolute insulin deficiency	Endogenous insulin secretion	*P*
*n*	5	167	
Age at diagnosis, years	63 (54–72)	58 (50–66)	0.28
Duration of diabetes, years	12 (9.5–19.5)	13 (9–17)	0.87
BMI, kg/m^2^	25.1 (22.8–28.8)	29.1 (26.3–33.6)	0.04
HbA_1c_, mmol/mol	72 (57–85)	62 (55–69)	0.24
HbA_1c_,%	8.7 (7.4–9.9)	7.8 (7.2–8.5)	
Time to insulin from diagnosis, years	2.5 (1.5–3)	6 (3–10.75)	0.005
Insulin/kg/24 h, units/kg/24 h	0.72 (0.54–0.88)	0.51 (0.31–0.84)	0.26
No. of subjects on oral hypoglycaemic agent, in addition to insulin (%)*	2/5 (40)	115/167 (69)	0.17
No. of subjects on basal‐bolus regime (%)*†	2/5 (40)	19/167 (11)	0.05

Data shown as medians (interquartile range).

*Chi‐squared tests; all others Mann–Whitney *U*‐test. †Basal‐bolus regime: four or five injections of insulin a day.

Two of the five participants with absolute insulin deficiency were GAD‐positive (titre in both >2000 units/ml); one of these was also IA2 positive (titre 74.9 units/ml). In addition, one patient who had two low UCPCR measurements from home but an sSCP of 0.37 nmol/l was GAD‐positive (titre >2000 units/ml). None of the nine participants from the comparison MMTT group, i.e. with home UCPCR demonstrating residual endogenous insulin secretion and confirmed on MMTT, were positive for GAD or IA2 antibodies.

Notably, only two of the five participants with absolute insulin deficiency were on a basal‐bolus regimen, and two were treated with oral agents in combination with insulin.

## Discussion

A total of 2.7% of insulin‐treated patients with a clinical diagnosis of Type 2 diabetes in the present study were found to have absolute insulin deficiency. Patients who may have had absolute insulin deficiency were detected using the simple non‐invasive testing method, the UCPCR, and a MMTT was used to confirm findings. These patients cannot be solely identified on the basis of clinical characteristics, or by testing of GAD antibodies.

### Prevalence and aetiology of absolute insulin deficiency in Type 2 diabetes

Our prevalence of absolute insulin deficiency of 2.7% (5/186) is similar to the 2.3% (3/133) found at 10 years from diagnosis in an observational study by Niskanen *et al*. 7. This looked at adult patients over the age of 45 years with new‐onset non‐insulin‐dependent diabetes, and measured sSCP and GAD titres at 0, 5 and 10 years. By including only insulin‐treated patients in our study, one might have expected a more insulin‐deficient group and hence a comparatively higher proportion of patients with absolute insulin deficiency than in the study by Niskanen *et al*. The aim for tighter glycaemic control (and hence earlier initiation of insulin) in the post‐Diabetes Control and Complications Trial/United Kingdom Prospective Diabetes Study era may provide an explanation for why this was not seen. Additionally, the 2.7% prevalence in our study population is a minimum: there were five additional participants with an initial UCPCR suggestive of absolute insulin deficiency who were either uncontactable or unable to undergo a MMTT (Fig. 1). If all these participants had confirmed sSCP <0.2 nmol/l, the prevalence would have risen to 5.2% (10/191).

In subjects with high titres of GAD antibodies and reasonably long diabetes duration (10–12 years), prospective longitudinal studies have shown that many (but not all) develop absolute insulin deficiency 7. When combined with the clinical features of adult‐onset diabetes not immediately requiring insulin treatment, the presence of pancreatic autoantibodies is known as LADA 7. Two of the five participants with absolute insulin deficiency in our study fit these criteria, having high GAD titres (>2000 units/ml, reference value >64 units/ml); however, with three participants with a confirmed absolute insulin deficiency not exhibiting GAD antibodies, it suggests that the presence of these antibodies is not a sensitive test for detecting the development of absolute insulin deficiency in those with long‐standing diabetes.

Our study has hence identified three people with apparent non‐autoimmune Type 2 diabetes and confirmed absolute insulin deficiency. Of the three patients developing absolute insulin deficiency in the study by Niskanen *et al*. 7, one was GAD‐antibody‐negative. This was the only other case we found in the literature of absolute insulin deficiency confirmed using sSCP in non‐autoimmune Type 2 diabetes 7. It is possible that the cross‐sectional measurement of pancreatic autoantibodies in our study may have missed some patients who were antibody‐positive at an earlier stage, but lost this positivity over time; however, numerous studies have found that high GAD titres persist 7. The cross‐sectional design of the present study meant we were able to look a wide range of durations of diabetes, longer than those looked at before in Type 2 diabetes, and this may help explain why we have detected absolute insulin deficiency where others have not. No previous studies we have found were designed to look for absolute insulin deficiency in Type 2 diabetes; the majority have looked at the significance of GAD antibodies on the deterioration in β‐cell function over time.

### Urinary C‐peptide creatinine ratio testing

The urinary C‐peptide creatinine ratio was used in this study as a practical test in a large number of individuals, and was able to detect patients at risk of absolute insulin deficiency. The gold standard MMTT was used to confirm findings. Those with evidence of endogenous insulin secretion on an initial UCPCR test had consistent results, both on repeat UCPCR and on MMTT. As would be expected by regression to the mean when selecting a low cut‐off, those with an initial low UCPCR suggesting absolute insulin deficiency had a tendency to higher results upon repeat testing, taking some above the designated 0.2 nmol/mmol threshold. In addition, some practical issues were identified which may have led to erroneously low UCPCR results on initial testing: these included patients tipping out the boric acid preservative from the sample pots, and postal delays. Additionally, in those with low endogenous insulin levels, variation in meal stimulus may have contributed to a low UCPCR on one occasion vs. a UCPCR over the 0.2 nmol/mmol threshold on another occasion. This is supported by the finding that, in four patients, despite two home UCPCR results suggestive of absolute insulin deficiency, a higher UCPCR and measurable (though low) sSCP levels were seen under controlled MMTT conditions. This suggests the MMTT was more stimulating than the home meals of these patients and they were still able to mount an insulin response when maximally stimulated. Nevertheless, insulin secretion with their normal diet may be more clinically relevant.

The screening method did identify individuals with genuine absolute insulin deficiency. With clear instructions on how optimally to take a sample for UCPCR testing, and advice to repeat a low UCPCR in the first instance, it is a very easy and practical test which has the advantage of being widely available, avoiding the need for venepuncture, and being able be carried out at home and posted in. Since the completion of the present study, it has been shown that the previously widely perceived practical limitations in measurement of C‐peptide in blood may be to some extent overcome by using ethylenediaminetetraacetic acid (EDTA) sample tubes: these can improve the stability of C‐peptide concentrations to > 24 h at room temperature (average 19.5°C) 21. This would also make measurement of C‐peptide in blood a viable test in the outpatient/primary care setting.

In the increasingly complex climate of diabetes management options, confirmation (or not) of insulin deficiency should help guide treatment, education and management decisions, which will be valuable in optimizing care for any patient, but perhaps particularly for the more frail, older patient. We would suggest that a measure of C‐peptide, such as UCPCR, may have an important role when clinical features, such as marked variation in blood glucose values, suggest absolute insulin deficiency.

### Clinical characteristics

In our study, those with confirmed absolute insulin deficiency had started insulin sooner after diagnosis than those with retained endogenous insulin (2.5 vs. 6 years), and had lower BMIs (25 vs. 29 kg/m^2^). In terms of other easily available and measurable baseline patient characteristics, there was little else to distinguish them.

Although two of the five patients with confirmed absolute insulin deficiency were on basal‐bolus regimens, the three others, and several of those with low endogenous insulin levels, were on unusual regimens more suited to patients with endogenous insulin secretion. Two of the five were still on oral hypoglycaemic agents, and none had had any training, such as the DAFNE course 12, to help them understand and manage their diabetes better.

Theoretically despite a clinical diagnosis of Type 2 diabetes, patients with absolute insulin deficiency may be at risk of the complications seen in Type 1 diabetes. This was reflected in all of the patients with absolute insulin deficiency – and those with low endogenous insulin levels ‐ reporting difficulty in managing their blood glucose levels owing to seemingly unpredictable fluctuations in blood glucose levels, and one patient having had an episode of ketoacidosis.

### Implications for clinical practice

Identification of absolute insulin deficiency in patients with a clinical diagnosis of Type 2 diabetes may enable optimization of their treatment such as basal‐bolus regimens, management and education such as DAFNE courses 12, and recognition of potential complications such as higher risks of hypoglycaemia or ketoacidosis. All these have not been traditional considerations in many patients with Type 2 diabetes, and recognition should help improve the quality of life of these individuals.

The UCPCR is a practical and useful test to detect absolute insulin deficiency in Type 2 diabetes and should be used in individuals with Type 2 diabetes developing ketoacidosis, severe hypoglycaemia or having a large fluctuation in blood glucose values, to help inform optimum diagnosis and/or management. A UCPCR suggestive of endogenous insulin production is reliable, and in this clinical context may suggest other explanations for the clinical features (such as compliance). A low UCPCR suggestive of insulin deficiency should be repeated in the first instance, but may help guide management and education as described above.

## Conclusion

In conclusion, we have shown that absolute insulin deficiency is present in 3% of insulin‐treated subjects with Type 2 diabetes and may be detected using UCPCR. Clinical features such as GAD antibodies, starting insulin sooner after diagnosis, and having a lower BMI are pointers to help recognize those at risk, but are not diagnostic. Those with absolute insulin deficiency are at risk of more fluctuant blood glucose levels, hypoglycaemia and ketoacidosis, which may adversely affect quality of life as well as potentially have more severe consequences, especially in the older population. Recognition of absolute insulin deficiency is thus important as it will aid the optimum management of these individuals, and the UCPCR is a useful test that can be used in general practice or in outpatients to confirm a clinical suspicion of insulin deficiency.

### Funding sources

This project was supported by the Peninsula National Institute for Health Research (NIHR) Clinical Research Facility, the Department of Health, and the Peninsula Collaboration for Leadership in Applied Health Research and Care (PenCLAHRC). A.T.H. is an NIHR and a Wellcome Trust senior investigator. A.T.H., B.A.K. and B.M.S. are supported by the NIHR Exeter Clinical Research Facility. NIHR have supported S.V.H. and A.G.J. through academic clinical fellowships, and support A.G.H. through a doctoral research fellowship. R.E.J.B was supported by Diabetes UK through a doctoral research fellowship. The views given in this paper do not necessarily represent those of NIHR, the NHS or the Department of Health.

### Competing interests

None declared.
